# Fracture Toughness of Winding Carbon Plastics Based on Epoxy Matrices and Reinforced by Polysulfone Film

**DOI:** 10.3390/polym17020220

**Published:** 2025-01-16

**Authors:** Eldar B. Dzhangurazov, Tuyara V. Petrova, Aleksey V. Shapagin, Ilya V. Tretyakov, Roman A. Korokhin, Aleksey V. Kireynov, Olga V. Alexeeva, Vitaliy I. Solodilov, Gleb Yu. Yurkov, Alexander Al. Berlin

**Affiliations:** 1 N.N. Semenov Federal Research Center for Chemical Physics, Russian Academy of Sciences, 119991 Moscow, Russiakorohinra@gmail.com (R.A.K.);; 2 NTI Center “Digital Materials Science: New Materials and Substances”, Bauman Moscow State Technical University, 105005 Moscow, Russia; alexkireinov@gmail.com; 3 Frumkin Institute of Physical Chemistry and Electrochemistry, Russian Academy of Sciences, 119071 Moscow, Russia; shapagin@mail.ru; 4 N.M. Emanuel Institute of Biochemical Physics, Russian Academy of Sciences, 119334 Moscow, Russia; alexol@yandex.ru

**Keywords:** carbon-fiber-reinforced plastics, epoxy oligomer, polysulfone film, crack resistance, fracture mechanism, cyclic load

## Abstract

In this work, the fracture mechanism of winding carbon-fiber-reinforced plastics (CFRPs) based on epoxy matrices reinforced by polysulfone film was investigated. Two types of polymer matrices were used: epoxy oligomer (EO) cured by iso-methyltetrahydrophthalic anhydride (iso-MTHPA), and EO-modified polysulfone (PSU) with active diluent furfuryl glycidyl ether (FGE) cured by iso-MTHPA. At the winding stage, the reinforcing film was placed in the middle layer of the CFRP. The fracture toughness *G_IR_* of the obtained CFRP was determined by the double-cantilever beam delamination method. Additionally, the effect of cyclic loading on the fracture toughness of CFRP reinforced with polysulfone film was investigated. It was shown that heterogeneous structures arising from the dissolution of the polysulfone film in the epoxy binder during the curing process increase the fracture toughness of CFRP from 0.5 kJ/m^2^ to 1.2 kJ/m^2^. Application of cyclic loads had little effect on the fracture toughness value. As a result of this study, it was revealed that the macrocrack propagates near the reinforcement layer along the diffusion zone, which has a phase organization of the type PSU matrix–EO dispersion.

## 1. Introduction

Epoxy matrices reinforced with high-strength fibers are widely used for the production of structural materials due to their combination of high strength, low density and corrosion resistance compared to metal structures [1,2]. Such materials are most often used for load-bearing structures in the construction of automotive, aerospace and marine equipment [3]. However, reinforced plastics based on an epoxy matrix have low fracture toughness, which can lead to premature destruction of the product or structure. This is primarily due to the fragility of the epoxy matrix, characterized by low plastic strains [4,5,6,7]. Many works are devoted to the study of increasing the fracture toughness of epoxy matrices by introducing various nature modifiers into them [8]. We can highlight some methods of strengthening epoxy resins using rubbers [9,10,11], thermoplastics [12,13,14], core-shell polymers [15,16,17], nanomaterials [18,19,20] and hyperbranched polymers [21,22,23].

The highest fracture toughness values of epoxy matrices and reinforced plastics based on them can be obtained by introducing heat-resistant thermoplastics [24,25,26,27,28]. This effect appears due to the formation of heterogeneous structures as a result of phase decomposition during curing of the mixed binder. The fracture toughness of such systems increases due to the dissipation of crack growth energy when interacting with thermoplastic-rich phases [29,30]. However, in reinforced plastics, there is a possibility that the thermoplastic-containing phase structure will not penetrate into the reinforced layers or will be distributed unevenly between the layers. Accordingly, when modified with thermoplastics, the resistance to crack growth in reinforced plastics will not be as effective as in non-reinforced matrices [31].

The literature has paid particular attention to the use of thermoplastic layers in the form of films [32,33,34,35,36,37] or nonwoven fiber materials [38,39,40,41] to strengthen reinforced plastics.

In [42], the maximum increase in the fracture toughness of G_IC_ carbon fiber/epoxy composite laminates by 61.5% (from 376.6 to 608.2 J/m^2^) was achieved by using polyethersulfone (PES) porous films (film thickness 97.5 μm) compared to composites without thermoplastic interlayers. The authors attributed that result to a cohesive failure mechanism, crack deflection and the formation of microcracks caused by PES microspheres. The use of uniformly aligned PES fiber webs in CFRP laminate also increased the fracture toughness of G_IC_ by 103% (from 376 to 765 J/m^2^) [43]. In that case, the improvement in interlaminar fracture toughness may be associated with the formation of characteristic interlaminar structures after phase separation in the PES-containing epoxy resin. The carbon fiber fabric coating with 20 wt.% poly(aryletherketone) (PAEK) increased the fracture energies of the carbon fiber G_IC_ from 313 to 647 J/m^2^ [44]. During the curing process of such a system, continuous layers with dispersed particles of epoxy oligomer (EO) enriched with PAEK were formed in the interlayer areas, which provides additional strengthening of the carbon fiber. A significant increase in fracture toughness was achieved in [45], where an increase in the strength of laminated carbon plastics was achieved by producing a two-component porous film consisting of PEK-C and PES of varying densities (from 16.9 to 24.1 gsm). The maximum G_IC_ increase of 135.3% (approximately from 0.7 to 1.6 kJ/m^2^) was obtained at a film density of 22 gsm. Due to the formation of “scale-like” and “sea-island-like” structures, the two-component PEK-C/PES films provided composite strengthening through shear yielding, crack pinning and crack deflection. As noted in the article [46], the efficiency of strengthening in epoxy-reinforced plastics using thermoplastic interlayers depends on the phase morphology formed in the cured matrix of the reinforced plastic. The authors used polyetherketone-cardo (PEK-C) film to strengthen CFRP. A significant improvement in fracture toughness was achieved by forming a gradient structure, which consisted of a “scale-like” structure, nodular morphology and a “sea-island” dual phase. Thus, the formation of a multi-phase heterogeneous structure provides an increase in the fracture energy due to the change and growth of the crack trajectory.

Nowadays, work on modifying epoxy systems with thermoplastics to increase the fracture toughness of reinforced plastics is developing in two directions. The first is the dissolution of thermoplastic in an epoxy binder before molding reinforced plastic, the second is the introduction of a layer of thermoplastic in the form of a film or non-woven material during the molding of the composite. However, to our knowledge, no comprehensive study has yet been conducted on the introduction of an additional thermoplastic layer into a reinforced plastic based on a hybrid epoxy resin containing dissolved thermoplastic.

This work continues our previous work on the production of low-viscosity epoxy binders containing thermoplastic [47,48,49]. The works [47,48,49] presented a systematic study of the fracture toughness and impact resistance of epoxy–polymer matrices and reinforced plastics based on them. It was shown that the phase structure of hybrid matrices of reinforced plastics determines their fracture toughness and impact resistance. The resistance to crack formation and the propagation of epoxy–polymer matrices and reinforced plastics based on them is determined by the length of the thermoplastic phases, as well as the degree of their involvement in the destruction process. Also obtained were correlations of the fracture toughness of hybrid matrices and reinforced plastics based on them. It can be stated that by increasing the fracture toughness of the epoxy matrix, the fracture toughness of reinforced plastics also increases, regardless of the type of thermoplastic polymers in the epoxy matrix. The present work aimed to study the influence of the resulting structure on the destruction process. We investigated, in contrast to the previously conducted work, how additional reinforcing layers of thermoplastic dissolve in the composite during the curing process and what structure is formed in the material. Two variants of CFRP matrices were proposed: unmodified (EO + hardener) and one modified with polysulfone and an active diluent (EO + PSU + FGE + hardener). According to the results of work [47], the modified matrix has a high fracture toughness (0.52 kJ/m^2^) compared to the unmodified one (0.17 kJ/m^2^), which is very promising for obtaining CFRP by traditional winding methods due to the introduction of the active diluent FGE into the composition. For each type of matrix, a reinforcing layer of polysulfone film was additionally provided in the middle layer of the CFRP. The polysulfone reinforcing film dissolves in the unmodified and modified matrices at different rates, which leads to the formation of gradient structures of different lengths. Thus, the fracture toughness of four types of wound CFRP was determined in this work. The dependence of the composite phase structure on its resistance to crack growth was established. The effect of cyclic loads applied to ring CFRP samples on their fracture toughness was studied. The obtained results were compared with morphological studies of the crack surface, and mechanisms of CFRP failure before and after exposure to cyclic loads were proposed. The conducted research will allow us to expand the approaches to strengthening reinforced plastics. This is especially applicable in the case of wound CFRP, characterized by the dense packing of monofilaments and the absence of pronounced layers in the reinforcing structure.

## 2. Materials and Methods

### 2.1. Materials

Two compositions based on epoxy binder were used for this work. The first composition consisted of epoxy oligomer (EO)—CHS EPOXY 520 resin (Spolchemie, Ustin nad Labem, Czech Republic), which was cured by anhydride-type iso-methyltetrahydrophthalic anhydride (iso-MTHPA (JSC CHIMEX Limited, St. Petersburg, Russia)) in the presence of accelerator 2-methylimidazole (2-MI (JSC CHIMEX Limited, St. Petersburg, Russia)).

The second type was epoxy oligomer CHS EPOXY 520 resin which was modified with active diluent furfuryl glycidyl ether (FGE (OOO “DOROS”, Yaroslavl, Russia)) and polysulfone (PSU) PSK-1 (AO “NIIPM”, Moscow, Russia) with a molecular weight of 35,000 g/mol. The quantitative ratio of the binder components is given below in Table 1.

Carbon roving UMT49-12K-EP (UMATEX, Moscow, Russia) was used for the reinforcing fibers. According to the manufacturer, the roving has a tensile strength of 4.9 GPa, elastic modulus of 260 GPa, elongation at break of 1.8%, density of 1.78 g/cm^3^ and linear density of 760 Tex, and the size is specified for epoxy binders.

### 2.2. Preparation of Binders

The unmodified binder was obtained by mechanically mixing the components shown in Table 1 (see the row for the unmodified binder) at room temperature.

Polymer mixtures were obtained by dissolving PSU in EO at a temperature of 100–120 °C. The ratio of the binder components is given in Table 1 (see the row for the modified binder). Then, at a temperature of 60–80 °C, the active diluent FGE was added to the mixture. Finally, the hardener iso-MTHPA and accelerator 2-MI were added to the resulting polymer mixture [47].

### 2.3. Preparation of Ring Samples of Carbon-Fiber-Reinforced Plastics with a Reinforcing Film in the Middle Layer

Unidirectional carbon fiber was produced by the filament winding method. The sample preparation technology is described in detail in [50] and is similar in implementation to the standard ASTM D2291/D2291M-16 [51]. Winding of two types of ring samples was performed, with and without (reference samples) a reinforcing layer of polysulfone film. The internal diameter for all rings was 150 mm.

At the first stage, half the thickness of the ring was formed. Then, a polysulfone film with a thickness of about 50 µm was placed into the middle layer of the samples. In the same layer, during winding, two PTFE films of 30 mm length and 10 mm width, which set the initial crack, were placed opposite each other in the middle part of the ring.

The reinforcing film was not placed inside the control CFRP samples. Finally, the second part of the ring was wound. The types and number of rings are shown in Table 2 and Table 3.

Composites were cured for 4 h at 140 °C. All the samples had the same technological and thermal background. The amount of reinforcing fibers, matrix and porosity were calculated based on the results of measuring their geometrical sizes and weight using the method described in [52]. The density of carbon fibers required for calculating fiber volume fractions and porosity was used according to the manufacturer’s data. The density of the polymer matrix was assumed to be 1.2 g/cm^3^. The density of carbon fiber reinforced plastics was determined according to ASTM D792-13 [53]. The calculation of the amount of reinforced plastic components showed that the fiber content was practically independent of the composition of the binder (55–65 vol.%), and the porosity did not exceed 2 vol.% (see Table 4).

**Figure 1:**
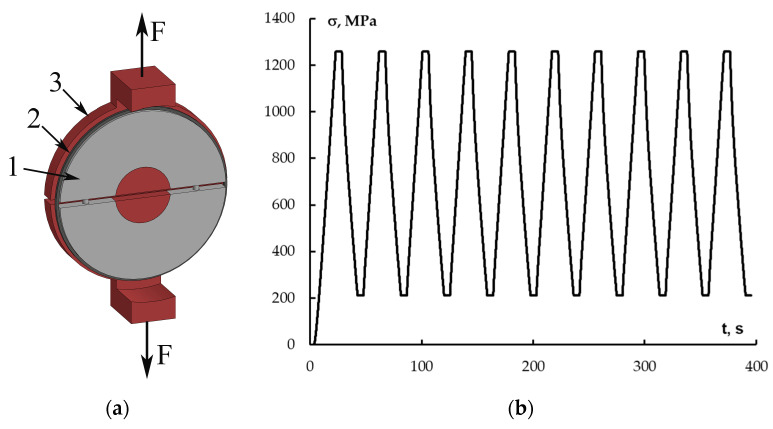
Tensile testing fixture (**a**) and cycles of stress application to ring-shaped CFRP samples (number of cycles 10) (**b**). 1—split disk, 2—ring sample, 3—supports for holding.

Rings with a thickness of 1.6 mm and a width of 10 mm were used to determine the tensile strength and modulus of the half discs. Control (reference) rings with an initial crack and rings reinforced with polysulfone film were divided into batches and subjected to cyclic loading. The rings were loaded according to the cycle shown in Figure 1. The numbers of cycles were 0, 1, 10 and 100. The range of applied stresses was in the range from 10% to 60% of the breaking stress.

**Figure 2:**
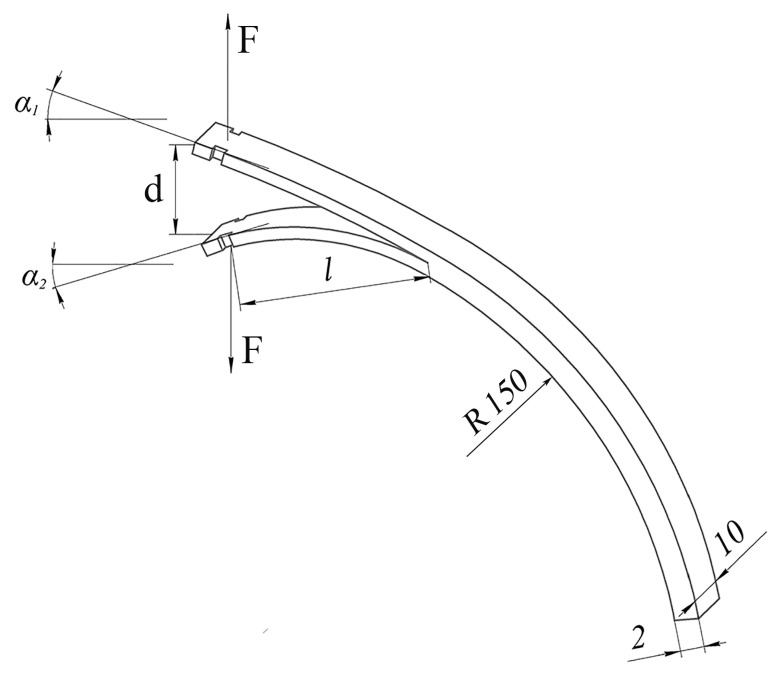
Scheme of a specimen for determining the crack resistance *G_IR_* of unidirectional fibrous composites.

The ring samples after the application of cyclic loads were cut into four parts so that the initial crack was in the end part of each specimen and had a length of 15 mm (Figure 2).

### 2.4. Characterization and Measurement

#### 2.4.1. The Test Method for Apparent Hoop Tensile Strength

The fracture stress and elastic modulus were determined on the obtained CFRP ring samples using the test method for apparent hoop tensile strength.

The experimental procedure was similar to that described in ASTM D2290-16 [54] for procedure A or the similar standard GOST 25.603-82 [55].

As noted in the standards, the defined value of the tensile strength may be slightly underestimated, since during the test a bending moment appears at the split-disk connector and stress concentration occurs. The CFRP samples were tested and cyclic loading was applied using a Zwick Z100 universal testing machine (ZwickRoell GmbH & Co., Ulm, Germany). The grips were moved at a speed of 10 mm/min. The force–displacement diagram was recorded during loading. For each type of material, 6 samples were tested. The apparent tensile strength at rupture of the specimen was calculated using the appropriate equation [54,55]:

(1) $$\sigma_{t}=F_{max}/2S,$$

where *F* is the applied load, and *S* is the average value of the initial cross-sectional areas of the sample in the middle of the measuring base.

The elastic modulus in the circumferential direction during tension E was calculated using Formula (2) [55]:

(2) E=ΔσΔε=ΔF2SΔε,

where Δ*F*—load increment; Δ*e* = Δ*l/l*—increment of relative deformation in the circumferential direction when the load changes by Δ*F*; *l*—initial length of the measuring base; and Δ*l*—increment of the initial length of the measuring base when the load changes by Δ*F*.

#### 2.4.2. Fracture Toughness

The crack resistance *G_IR_* of reinforced plastics was determined using the “angle method” [48]. This method makes it possible to determine the interlayer fracture toughness of *G_IR_* composites on the specimens in the form of ring segments and is applicable to both weak and strong bending of cantilevers. The tests were carried out on an Instron 3365 (INSTRON Company, Norwood, MA, USA) test machine. The displacement speed of the grips was 50 mm/min. During the tests, the relation between the force *F* and the displacement *D* of the grips was recorded. From loading diagrams, the force *F* was determined, which was necessary to calculate the energy *G_IR_*. Photographs were used to measure the bending angles of cantilevers of the specimens. Additionally, after each increase in crack length, the total crack length was measured.

The specific fracture toughness (crack resistance *G_IR_*) was calculated by the formula:

(3) $$G_{IR}=F(\sin\alpha_{1}+\sin\alpha_{2})/b$$

where *F* is the force at which the crack begins to move; *α*_1_ and *α*_2_ are the bending angles of the cantilevers; and *b* is the specimen width.

The number of loading–unloading cycles during testing of each specimen varied from 4 to 6.

#### 2.4.3. Electron Microscopy

The fracture surface morphology of the delaminated specimens and their cross-sections was investigated using a Phenom ProX scanning electron microscope with EDX detector (Thermo Fisher Scientific, Waltham, MA, USA). The surface of the samples was studied in the backscattered electrons mode. Two types of samples were studied. The first type was a cross-section of CFRP. To identify the features of the formed structure of the modified polymer matrix, the surface of the section was treated with methylene chloride vapors, which partially dissolved the polysulfone. The second type of samples was the surface of a crack formed after delamination of a sample of a double-cantilever CFRP beam.

## 3. Results and Discussion

### 3.1. Tensile Properties of CFRP Ring Samples

The tensile strength σ and elastic modulus E of the CFRP ring samples are given in Table 5. It is shown that for CFRP based on the unmodified binder and not containing a reinforcing layer, the value of the elastic modulus (267 GPa) is comparable to the value E declared by the manufacturer of the carbon fibers (260 GPa). Incorporation of a reinforcing layer into the middle section of the CFRP ring samples leads to a reduction in the elastic modulus to 230 GPa.

An increase in the elastic modulus of CFRP ring samples to 268 GPa was achieved by using a modified binder. In this case, the introduction of the reinforcing layer reduced the elastic modulus of the CFRP to 259 GPa. It should be noted that the introduction of a strengthening layer led to changes in the elastic modulus for the CFRP based on an unmodified matrix by no more than 14%; for the CFRP based on an epoxy polymer matrix, it was 6%.

A similar trend was observed for the tensile strength when introducing a reinforcing layer into the CFRP. If unmodified binder was used as the CFRP matrix, then the introduction of a reinforcing layer changed the tensile strength parameters from 2.09 GPa to 1.88 GPa.

In the case of CFRP based on the modified binder, the change in tensile strength of the ring samples was less obvious: 2.21 GPa without the reinforcing layer and 2.12 GPa with the reinforcing layer. The mechanisms of change in the tensile strength and elastic modulus of the CFRP with the introduction of a reinforcing layer will be discussed below. The obtained tensile strength values were used to calculate the loads under cyclic loading of CFRP ring samples.

### 3.2. Fracture Toughness of CFRPs

**Figure 3:**
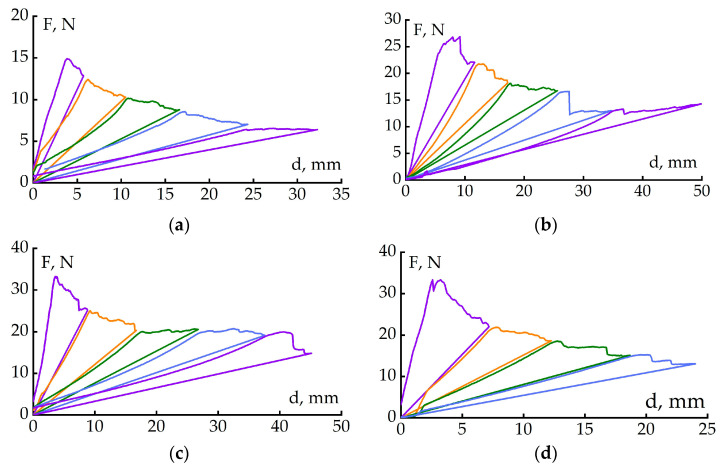
Loading diagrams of CFRP control samples (without cyclic loading) based on EO+iso-MTHPA+2-MI (**a**), EO+iso-MTHPA+2-MI+PSU film (**b**), EO+PSU+FGE+iso-MTHPA+2-MI (**c**) and EO+PSU+FGE+iso-MTHPA+2-MI+PSU film (**d**).

Figure 3 shows delamination diagrams of the CFRP double-cantilever beams. All the CFRP samples that were delaminated for the first time, before reaching the critical force or the maximum load at which the crack grows, were deformed elastically. After reaching the maximum force, the crack grows, which is accompanied by load fluctuations. Load fluctuations indicate that the rate of crack propagation is not constant. After the crack had grown to a predetermined length, the sample was unloaded. Then, the cycle of loading—crack growth—unloading was repeated several times.

The crack propagation characteristics for CFRP with and without the PSU reinforcing layer, as well as after cyclic loading, can be assessed from Figure 3a. It can be seen that the section of the diagram describing crack propagation in the CFRP based on the unmodified matrix is characterized by relatively small changes in load during crack propagation. The introduction of the reinforcing PSU film into the CFRP middle layer leads to noticeable load fluctuations during crack propagation, i.e., the crack grows with abrupt extensions (Figure 3b). Such crack propagation is probably due to the heterogeneity of the material and the change in the crack level during its extension.

Also noted in Figure 3c are the load variations during crack propagation in the modified matrix-based CFRP. These load variations during crack growth are comparable to those observed for the unmodified matrix-based CFRP with a reinforcing layer.

Load surges also accompany the crack propagation process if a reinforcing layer is introduced into the CFRP based on a modified matrix (Figure 3d).

**Figure 4:**
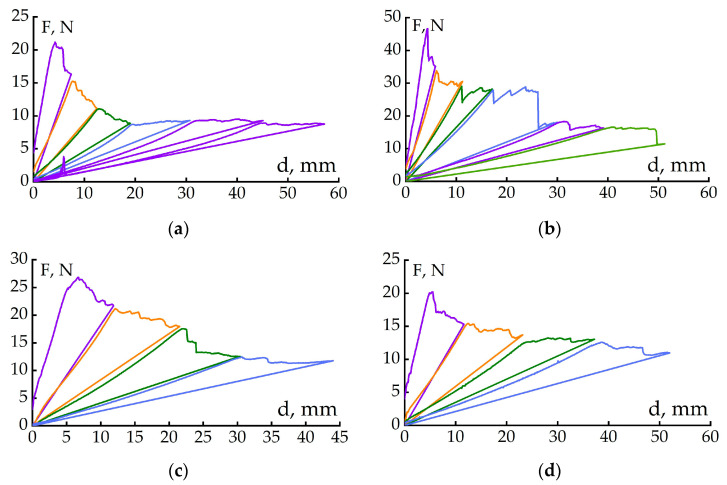
Loading diagrams of CFRPs based on the binder EO+iso-MTHPA+2-MI (**a**), EO+iso-MTHPA+2-MI+PSU film (**b**), EO+PSU+FGE+iso-MTHPA+2-MI (**c**) and EO+PSU+FGE+iso-MTHPA+2-MI+PSU film (**d**), after applying 100 load–unload cycles.

The CFRP loading diagrams after 100 loading–unloading cycles are shown in Figure 4. After analyzing the results, we can conclude that regardless of the type of material and the presence of a reinforcing layer in the middle layer, the crack propagation pattern does not change. Figure 4a shows smooth crack propagation for unmodified CFRP without reinforcing PSU film. In other cases (Figure 4b–d), the crack in the CFRP grows unstably and a noticeable drop in load is observed upon elongation. The crack propagation mechanism for all types of CFRP will be discussed below.

**Figure 5:**
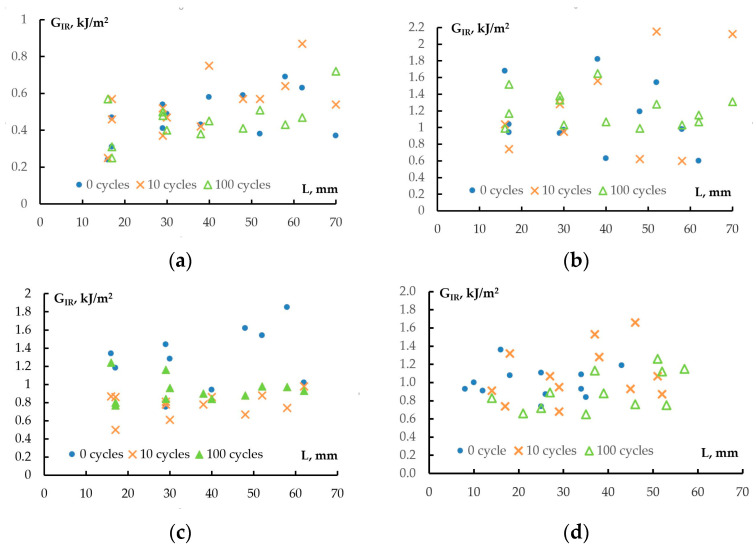
Changes in fracture toughness values of *G_IR_* CFRPs based on EO+ iso-MTHPA+2-MI (**a**), EO+iso-MTHPA+2-MI+PSU film (**b**), EO+PSU+FGE+iso-MTHPA+2-MI (**c**) and EO+PSU+FGE+iso-MTHPA+2-MI+PSU film (**d**), with crack length *L*. The figures show the number of load–unload cycles.

The fracture toughness *G_IR_* is calculated from the loading diagrams that correspond to each crack extension. The changes in *G_IR_* versus crack length are shown in Figure 5.

Figure 5a shows that the *G_IR_* values for CFRP based on the unmodified matrix increase with increasing crack length. The application of a cyclic load does not affect the specific changes in fracture toughness with increasing crack length. The increase in *G_IR_* values is associated with the formation and propagation of strands [56,57].

There is no clear dependence of the crack resistance *G_IR_* on the crack length L when introducing a PSU film reinforcing layer into CFRP (Figure 5b). A significant spread of *G_IR_* values (0.6–1.8 kJ/m^2^) could be attributed to the significant heterogeneity of the material. The greater the number of applied load–unload cycles, the more the cloud of *G_IR_* values narrows. Thus, for 100 load-unload cycles, the range of fracture toughness changes from 1 to 1.6 kJ/m^2^.

For the CFRP based on the modified matrix (Figure 5c), an increase in fracture toughness with increasing crack length is also characteristic. However, under cyclic loading, fracture toughness is practically independent of the crack length, and the *G_IR_* value decreases from ~1.2 kJ/m^2^ to 0.8 kJ/m^2^. For the modified CFRP with a reinforcing layer, no clear dependence of fracture toughness versus crack length is observed (Figure 5d). The *G_IR_* values vary from 0.6 to 1.2 kJ/m^2^. The average fracture toughness values are shown in Table 6.

The fracture toughness of the control series of CFRPs did not change with the increasing number of applied cycles and was about 0.5 kJ/m^2^. The spread of data did not exceed 0.1 kJ/m^2^. The introduction of a polysulfone film into the middle layer of carbon fiber led to an increase in the fracture toughness of samples to 0.91 kJ/m^2^ without cyclic loading, and with cyclic loading, an increase in the *G_IR_* values to 1.2 kJ/m^2^ was observed. The scatter of *G_IR_* data for CFRP reinforced with polysulfone film was 0.34 kJ/m^2^. Compared to unmodified CFRP, the scatter of values increased threefold, which was probably due to the heterogeneity of the material. After applying 100 load–unload cycles to the CFRP with a reinforcing layer, a twofold decrease in the scatter of *G_IR_* values to 0.17 kJ/m^2^ was observed. It is possible that microdamage accumulation was observed in the material, which leads to the localization of the macrocrack path.

The use of a polysulfone-modified epoxy matrix allows increasing the fracture toughness of CFRP from 0.5 to 1.25 kJ/m^2^. Such an increase in fracture toughness is associated with the formation of heterogeneous structures during the curing of the blended binder, which we discussed earlier in [48]. The extended phase of the thermoplastic significantly increases the energy for its propagation due to its microplasticity and involvement in the crack formation process. The heterogeneity of the material is also evidenced by the large scatter of data, which reaches 0.29 kJ/m^2^. When applying a cyclic load, a decrease in fracture toughness to 0.8–0.9 kJ/m^2^ is observed, and the scatter of data decreases to 0.1 kJ/m^2^.

The introduction of a reinforcing layer into the CFRP middle layer based on a modified matrix reduces the fracture toughness to 1 kJ/m^2^. Such a reduction seems unexpected. Apparently, in this case, the growing crack propagates near the reinforcing layer along structures that provide lower fracture toughness. The application of cyclic loading reduces the fracture toughness of the CFRP to approximately 0.9 kJ/m^2^. It should be noted that the spread of *G_IR_* values in this case is higher than for the modified CFRP and does not depend on the number of applied cycles. This may indicate significant heterogeneity of the material and random crack propagation near the reinforcing layer.

### 3.3. Structural and Morphological Study

**Figure 6:**
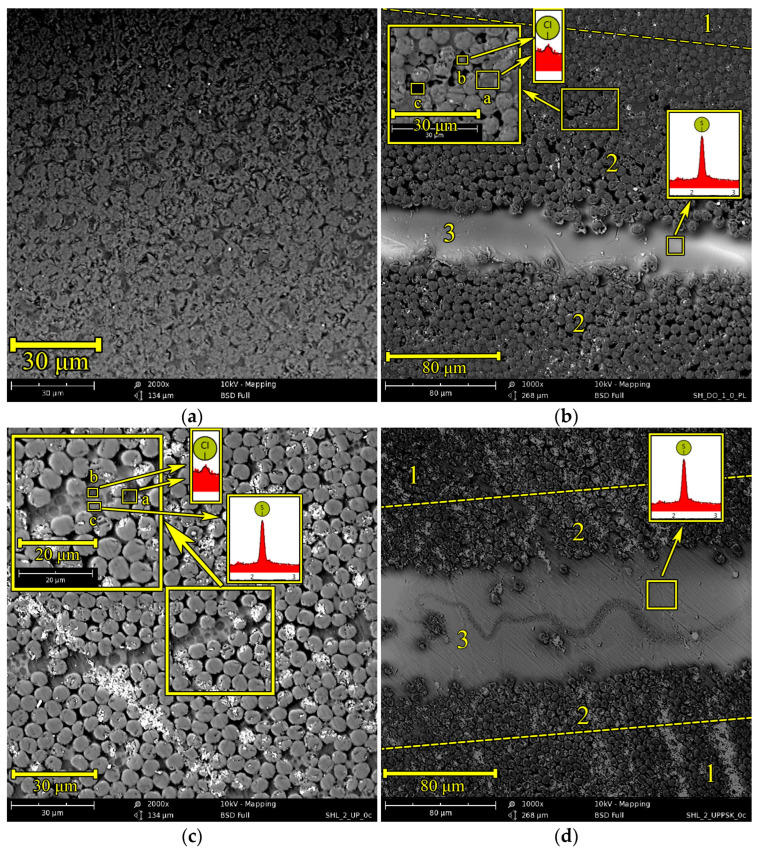
SEM micrographs of cross-sections of CFRPs based on EO+iso-MTHPA+2-MI (**a**), EO+iso-MTHPA+2-MI+PSU film (**b**), EO+PSU+FGE+iso-MTHPA+2-MI (**c**) and EO+PSU+FGE+iso-MTHPA+2-MI+PSU film (**d**).

Figure 6 shows cross-sections of CFRP. The control CFRP sample (Figure 6a) is characterized by a dense packing of carbon monofilaments with a diameter of about 6 μm. The distance between the monofilaments is about 1 μm.

The cross-section of the CFRP based on an unmodified matrix and containing a reinforcing layer (Figure 6b) consists of three regions. Region 1 is characterized by a dense packing of reinforcing carbon fibers and represents the reinforcing structure of the carbon-fiber-reinforced plastic. As a consequence of the dissolution of the polysulfone film in the epoxy oligomer matrix, the density of the reinforcing structure becomes lower—in region 2. Study of the phase structure in the interfiber space of region 2 after etching the surface layer with methylene chloride showed that the exposed structure belongs to the matrix-dispersion type with an epoxy-enriched dispersed phase. According to the EDX analysis, the characteristic X-ray spectrum of region “a” contains the Кα line of chlorine. The presence of chlorine in region “a” confirms that the dispersed phase is enriched in EO (DGEBA-based). The particles in region “b” are also characterized by a peak corresponding to the Kα line of chlorine. Region “c” is characterized by the free volume formed during the treatment of the sample surface with methylene chloride. Summarizing the above, it can be stated that the dissolution of PSU in region 2 confirms the enrichment of the continuous phase with thermoplastic. In Figure 6, this region is identified by dark areas formed after treatment with methylene chloride. The farther from the reinforcing film layer, the more complex the structure becomes. Regions with dimensions of 15–30 μm of the PSU matrix—EO dispersion type are formed. The dispersed phase size is 1.5–6 µm. At an even greater distance, an epoxy matrix with PSU inclusions less than 1 μm in size is observed (identified in dark areas by “voids” of the corresponding size in Figure 6).

The heterogeneity of the structure, due to the presence of an EO-enriched phase and a PSU-enriched phase, was also confirmed in our earlier studies [47]. When studying the matrices using the DMA method, the presence of two tgα peaks was detected, which correspond to the glass transition temperature of the EO-rich phase and the PSU-rich phase. In this case, when the composition of the mixed composites changes, the position of the peaks on the temperature scale changes. The DSC method does not allow identifying the presence of two or more phases in epoxy–polymer matrices, since the method itself allows recording the integral characteristic of the sample. It should be noted that the heterogeneous structures of the epoxy–polymer binders formed during the curing process in the free and constrained volume of reinforcing fibers have different phase organization. The phase organization of such structures is described in detail in [48,49]. It is shown that in enriched PSU-reinforced plastics a smaller number of extended structures are formed than in an unlimited volume. However, the preservation of the effect of increasing fracture toughness for hybrid matrices when reinforced with continuous fibers depends on the degree of involvement of heterogeneous structures in the process of crack formation.

The formation of this type of phase structure in the interfiber space, coupled with the absence of phase formations in the reinforcing layer 3, indicates the predominance of the rate of diffusion processes of polysulfone into the epoxy oligomer over the diffusion of the oligomer into the polysulfone matrix, as we showed earlier in [58]. The size of the epoxide-enriched dispersed phases reaches 3 μm, which is conditioned by the high diffusion mobility of the oligomer at the stage of phase particle formation. Note that a similar inverted phase structure in the EO–PSU concentration-gradient system according to the data of [59] is observed in the range of PSU concentrations from 20 to 80 wt.%. In the cured system, on each side of the PSU film (region 3), the width of region 2 is approximately 100–150 μm. The thickness of film 3 is approximately 30–45 μm.

The CFRP reinforcing structure based on the modified matrix also has a high packing density of reinforcing fibers (Figure 6c). In areas where the distance between reinforcing fibers is less than 1 µm, phase structures enriched with epoxy oligomer are observed. EDX analysis in the “a” region showed the presence of chlorine in the spectrum, which identifies this region as an epoxy phase. In places where the distance between the reinforcing fibers is more than 5 μm, phase structures of 10–40 μm in length with the matrix-dispersion phase organization are observed. The dispersed phase, enriched with EO, has a size of 0.5–3 μm. The phases of the described structure were identified using EDX. In the “c” region, the characteristic Kα-line of sulfur was identified on the X-ray spectrum, which confirms the enrichment of this phase with PSU. The presence of chlorine in the “b” region indicates the enrichment of the dispersed phase with EO.

Figure 6d shows a cross-section of CFRP containing a reinforcing layer of polysulfone film. It is evident that the dissolution of the reinforcing layer occurred more slowly during matrix curing, which is due to a decrease in the concentration gradient in the PSU film—EO + PSU system, which is the driving force of diffusion processes. Here, the thickness of the polysulfone film is about 80 μm, and the width of the diffusion zones is about 30 μm. In the diffusion zones, structures of the PSU matrix–EO dispersion type with a size of 3–10 μm were found, which exceeds the sizes of epoxy-enriched dispersed phases when using an unmodified binder. This is explained by a decrease in the rate of the chemical curing reaction when using a thermoplastic modifier dissolved in it. As a consequence, the system spends a long time in the process of phase decomposition at lower degrees of conversion, which leads to greater mass transfer of the substance to the nucleation centers.

As described above, the introduction of a reinforcing layer into CFRP can lead to a decrease in the elastic modulus and tensile strength. First of all, this is a consequence of the disruption of the reinforcing structure. Apparently, a sufficiently thick interlayer of the reinforcing layer makes the ring sample work as two parts. Here, the tension during ring stretching will be transmitted through the reinforcing layer, since the adhesion of epoxy resins and thermoplastics, as well as their mixtures, to fibers is comparable. In some cases, the use of epoxy–polymer mixtures helps to increase the adhesive strength [60]. Considering the greater deformability of the thermoplastic layer than the epoxy matrix, it can be assumed that the efficiency of stress transfer from one part of the ring to another will be lower. Creep of the reinforcing layer can also affect stress transfer. Additional studies will be required to identify more precisely the mechanism of the destruction of ring samples under tension. A decrease in the volumetric filling with fibers and an increase in the interfiber distance when dissolving the polysulfone film in carbon-fiber-reinforced plastic based on an unmodified matrix leads to a decrease in the elastic modulus by 8% and in the tensile strength by 10% compared to the control samples. For CFRP samples based on a modified binder, due to the preservation of the reinforcing structure, the decrease in strength characteristics is not so noticeable—less than 5%—and the decrease in strength characteristics is comparable to the scatter of data.

**Figure 7:**
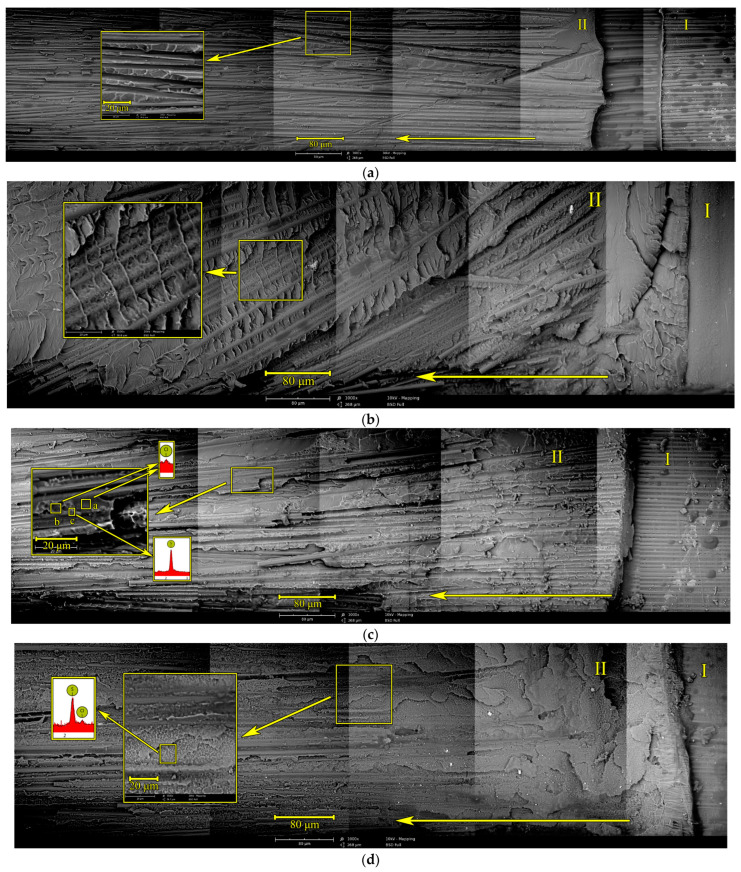
SEM micrographs of the delamination surface panoramas of CFRPs based on EO+ iso-MTHPA+2-MI (**a**), EO+iso-MTHPA+2-MI+PSU film (**b**), EO+PSU+FGE+iso-MTHPA+2-MI (**c**) and EO+PSU+FGE+iso-MTHPA+2-MI+PSU film (**d**). The number of applied load–unload cycles was 100.

Figure 7 shows micrographs of the delamination surface of CFRP.

Figure 7a shows a panorama of the crack surface of unmodified CFRP. Region I corresponds to the location of the initial crack and region II to the propagation of the macrocrack, which is of the greatest interest when considering the mechanisms of crack propagation. Delamination of CFRP in region II occurs predominantly along the polymer matrix—reinforcing fiber interface. The unmodified epoxy matrix undergoes brittle fracture. It is important that the failure mode of CFRP does not change after applying 100 load cycles. Fracture toughness remains unchanged—about 0.5 kJ/m^2^.

We have already noted above that a gradient structure is formed during the curing process. From Figure 7b, it is evident that CFRP delamination occurs with significant plastic microdeformations of the matrix. At the same time, experiencing the resistance of plastic structures, macrocracks try to pass through the reinforcing fibers, which leads to their destruction. Deeper in the material, the polymer matrix is destroyed along the diffusion layer, the structure of which can be described as PSU matrix–EO dispersion. The propagation of a crack through such a structure is characterized by a fracture toughness value of about 1 kJ/m^2^ (see Table 6). The *G_IR_* values here have a significant scatter of ±0.3 kJ/m^2^, which is explained by the complex path of the crack and multiple intersections of the gradient polymer matrix, and the involvement of reinforcing fibers in the destruction process. The increase in fracture toughness (up to 1.2 kJ/m^2^) after application of cyclic loading seems unexpected in the conditions of unchanged fracture surface morphology. Such an increase in *G_IR_* values can probably be related to stress relaxation in CFRP.

Table 6 shows that the CFRP based on the modified matrix has the highest fracture toughness—1.25 kJ/m^2^. In our previous works [36,41,45,47,48,50], it was noted that the high fracture toughness of reinforced plastics is determined by the heterogeneous structure of the matrix and the involvement of these structures in the crack growth process. Figure 7c shows that the propagation of the macrocrack is accompanied not only by delamination along the matrix–fiber boundary but also shows extensive areas of cohesive failure (along the matrix). Fragments of the matrix are visible on the reinforcing fibers, and the failure of the matrix is elastic–plastic. Areas with microplastic destruction are typical for extended structures of the PSU matrix–EO dispersion type. The length of these areas fluctuates within 10–500 µm. Involvement of such structures in the destruction process due to their high microplasticity increases the energy required for crack propagation. The decrease in *G_IR_* values to 0.8–0.9 kJ/m^2^ under cyclic loads is probably due to the accumulation of microcracks. The cause of the accumulation and development of such microdamages requires a separate study. An important fact is that even after applying cyclic loads to CFRP based on a modified matrix, its fracture toughness is almost twice as high as for unmodified CFRP.

Additional strengthening of CFRP with a polysulfone film based on a modified matrix did not allow a significant increase in fracture toughness (see Table 6). The *G_IR_* value was comparable to CFRP without a polysulfone film. Figure 7d shows that not only the film dissolution areas, but also the reinforcing layer itself, participates in the fracture process; the growing crack repeatedly passes through the polysulfone film. The structure near the reinforcing layer can be characterized as a PSU matrix – EO dispersion. Moreover, this structure is homogeneous over the entire surface of the resulting macrocrack. The application of cyclic loading does not significantly change the fracture toughness values of such a material, such as for CFRP based on a modified matrix. The *G_IR_* value remains at the initial value and is about 0.9 kJ/m^2^. Taking into account the scatter of data, it can be argued that the fracture toughness of modified CFRP reinforced with polysulfone film practically does not change when cyclic loads are applied, in contrast to the CFRP based on a modified matrix, where a decrease to 0.8 kJ/m^2^ is observed. Thus, the introduction of an additional strengthening layer, even in materials with high initial fracture toughness, allows them to maintain their properties at a high level after cyclic loading.

## 4. Conclusions

The promising potential of using polysulfone film as a reinforcing layer in winding CFRP is shown. The introduction of polysulfone film into the middle layer of a CFRP based on an unmodified epoxy matrix increases the *G_IR_* values from 0.5 kJ/m^2^ to 0.9 kJ/m^2^. Using this method of strengthening, it is possible to achieve *G_IR_* values comparable with the fracture toughness of a CFRP based on an epoxy polysulfone matrix (1.25 kJ/m^2^). An attempt to further strengthen CFRP based on a modified matrix leads to a slight decrease in fracture toughness to 1 kJ/m^2^. SEM studies have shown that in all cases the increase in fracture toughness is associated with the formation of heterogeneous structures that form during the phase decomposition of epoxy–polysulfone mixtures or the dissolution of the polysulfone film during curing. In the middle layer of the CFRP, gradient structures of the matrix-dispersion type with an epoxy-rich dispersed phase are formed during the dissolution of the PSU film. In the modified epoxy polysulfone matrix, phase decomposition and growth of heterogeneous structures occur during the curing process. Heterogeneous structures with extended thermoplastic phases are almost always much more resistant to the initiation and propagation of cracks. At the same time, it can be considered that the main contribution to the increase in the crack resistance of hybrid matrices is made by the mechanism for implementing the microdeformability of extended thermoplastic phases. Thus, the more microplastic phases of polysulfone in the composite matrix are involved in the destruction process, the higher the fracture toughness of the material. The highest fracture toughness values are obtained for CFRPs where delamination occurs along the PSU matrix–EO dispersion type structures. The heterogeneity of the obtained structures can be estimated from the scatter of the *G_IR_* data. For CFRPs based on a homogeneous unmodified matrix, the scatter of the *G_IR_* data is about ±0.1 kJ/m^2^. For a CFRP the matrix of which is heterogeneous or gradient structures, the scatter of the *G_IR_* values is ±0.15–0.35 kJ/m^2^.

The application of cyclic loading to the CFRP ring samples leads to changes in the *G_IR_* data (see Table 6). The most noticeable changes in fracture toughness after 100 loading–unloading cycles are observed for CFRPs based on the modified matrix (from 1.25 to 0.94 kJ/m^2^). In other cases, the *G_IR_* data remain almost unchanged and exceed the fracture toughness value for the control CFRP by two times. The change in the fracture toughness of CFRP upon the application of cyclic loads may be associated with the accumulation of microcracks and relaxation of residual stresses. These two mechanisms may compete, depending on the matrix structure. Microcrack accumulation in heterogeneous and graded matrices may not be as critical as for homogeneous ones. The initially high fracture toughness of hybrid epoxy–polymer matrices will effectively inhibit the formation of microcracks, as well as prevent their subcritical growth due to the PSU-enriched microplasticity of the phases. The values of fracture toughness *G_IR_* will change insignificantly. Also, due to the microplasticity of the polymer phase, in the case of the relaxation of residual stresses in CFRP, an increase in fracture toughness can be observed. The assumptions expressed deserve special attention and clarification of the destruction mechanisms.

The modulus of elasticity and tensile strength decrease by 14% for CFRP based on an unmodified matrix when introducing a reinforcing layer, and by 6% for CFRP based on an epoxy polymer matrix. Therefore, the use of hybrid matrices is promising not only for the purpose of regulating the phase structure of the matrix, but also for a small change in elastic-strength properties under tension.

The possibility of creating materials with a high level of fracture toughness under cyclic loads is opening up due to gradient reinforcing layers in the reinforcing structure of reinforced plastics. The proposed method of reinforcement will allow the strengthening of composite structures in critical areas, preventing their premature destruction. The combined use of reinforcing layers and hybrid matrices makes it possible to further expand the possibilities for regulating the fracture toughness of reinforced plastics and developing materials with the required set of properties for specific conditions.

## Figures and Tables

**Table 1:** Ratio of components in unmodified and modified epoxy binder.

Type of Binder	Components, mas.%				
	EO	PSU	FGE	iso-MTHPA	2-MI
Unmodified	100	0	0	90	0.2
Modified	100	38	38	124	0.2

**Table 2:** Nomenclature of CFRP rings wound on the basis of unmodified and modified binders for determining tensile strength properties.

Binder	EO+iso-MTHPA+2-MI (Unmodified)		EO+PSU+FGE+iso-MTHPA+2-MI (Modified)	
Composition	CFRP	CFRP+PSU Film	CFRP	CFRP+PSU Film
Number of rings	6	6	6	6
Number of samples	6	6	6	6

**Table 3:** Nomenclature of CFRP rings wound on the basis of unmodified and modified binders for determining crack resistance *G_IR_*.

Binder	EO+iso-MTHPA+2-MI (Unmodified)								EO+PSU+FGE+iso-MTHPA+2-MI (Modified)							
Composition	CFRP				CFRP+PSU Film				CFRP				CFRP+PSU Film			
Cycles	0	1	10	100	0	1	10	100	0	1	10	100	0	1	10	100
Number of rings	1	1	1	1	1	1	1	1	1	1	1	1	1	1	1	1
Number of samples	4	4	4	4	4	4	4	4	4	4	4	4	4	4	4	4

**Table 4:** Density, porosity and fiber content.

Binder	EO+iso-MTHPA+2-MI (Unmodified)		EO+PSU+FGE+iso-MTHPA+2-MI (Modified)	
Composition	CFRP	CFRP+PSU Film	CFRP	CFRP+PSU Film
ρ, g/cm^3^	1.60	1.59	1.61	1.60
V_f_, vol.%	65	60	60	55
V_porous_, vol.%	1	2	2	2

**Table 5:** Tensile strength *σ* and elastic modulus *E* of CFRP.

Binder	EO+iso-MTHPA+2-MI		EO+PSU+FGE+iso-MTHPA+2-MI	
Composition	CFRP	CFRP+PSU Film	CFRP	CFRP+PSU Film
*E*, GPa	267 ± 2	230 ± 7	268 ± 8	259 ± 12
*σ*, GPa	2.09 ± 0.07	1.88 ± 0.10	2.21 ± 0.08	2.12 ± 0.17

**Table 6:** Fracture toughness *G_IR_* (kJ/m^2^) of carbon fiber reinforced plastics after cyclic loading.

Binder	EO+iso-MTHPA+2-MI		EO+PSU+FGE+iso-MTHPA+2-MI	
Material	CFRP	CFRP+PSU Film	CFRP	CFRP+PSU Film
0 cycles	0.49 ± 0.10	0.91 ± 0.34	1.25 ± 0.29	1.00 ± 0.13
1 cycle	0.50 ± 0.07	1.23 ± 0.34	0.80 ± 0.18	1.31 ± 0.16
10 cycles	0.56 ± 0.10	1.23 ± 0.28	0.78 ± 0.10	1.08 ± 0.24
100 cycles	0.49 ± 0.06	1.21 ± 0.17	0.94 ± 0.10	0.90 ± 0.18

## Data Availability

The original contributions presented in this study are included in the article material. Further inquiries can be directed to the corresponding authors.
